# Managing Complex Foot Crush Injuries: A Case Report

**DOI:** 10.7759/cureus.52572

**Published:** 2024-01-19

**Authors:** Palak R Ahuja, Aditi Akhuj, Vaishnavi Yadav, Purva Gulrandhe, Aditi P Ambekar

**Affiliations:** 1 Department of Cardiovascular and Respiratory Physiotherapy, Ravi Nair Physiotherapy College, Datta Meghe Institute of Higher Education and Research, Wardha, IND

**Keywords:** cuneiform fracture, cuboid fracture, dyspnea, strength, rehabilitation, crush injuries

## Abstract

A serious kind of fractured foot ailment is a foot crush injury. Foot injury commonly happens in accidents involving transportation or the workplace, such as automobile accidents, big objects falling on the foot, or heavy machinery running over the foot. Foot crush injuries are more severe than regular foot fractures. These wounds are usually very serious, involving many fractures and soft tissue injuries. The main symptoms include pain, severe muscle and tissue damage, and extreme swelling. Because of this, treating a foot crush injury can be quite challenging and frequently requires the collaboration of physical therapists, orthopedic surgeons, and podiatrists. Physiotherapy is important for reducing pain, increasing range of motion, strengthening muscles, and improving leg function. It also decreases the chance of contractures, deformities, and stiffness following crush injuries. In this report, we present the case of a 58-year-old male with a lacerated wound over his left foot with chief complaints of severe pain. Patient-tailored physiotherapy rehabilitation, including active movements, passive movements, isometric exercises, and a strengthening regimen consisting of numerous repetitions and progressive complexity, was given. At the end of four weeks, the patient had improved strength and quality of life.

## Introduction

Foot crush injury is a severe type of broken foot condition. Crush injuries are any consequences of the body being compressed, such as those resulting from cave-ins, collapsed buildings, mine disasters, and earthquakes [1]. Living in the modern era entails coping with the growing nature of contemporary conflict and international terrorism in addition to natural and man-made disasters [2]. Man has created weapons of mass destruction, which, when used by warring nations or urban terrorists, can result in explosions, building collapses, and other events that induce a distinctive pattern of damage to the human body called crush injuries. Despite the immediate mortality, they can result in extensive complex hemodynamic and metabolic dysfunction over time in the survivors, which calls for attentive medical treatment [3]. There are two types of crush injuries: crush syndrome, which is a systemic injury, and compartment syndrome, which is a localized injury [4]. Crush injury symptoms include fractures, lacerations, bruising, pain, edema, and numbness [5].

Every year, millions of people throughout the world experience natural or man-made disasters, which can result in mass casualty situations and serious medical conditions such as crush injury and syndrome [6]. Foot trauma symptoms depend on the severity of trauma. Crush injuries are characterized by pain, bruising, and swelling ranging from mild to severe [7]. The lower extremities are most frequently affected by direct physical trauma and compression that cause a crush injury to the human body. Asphyxia, severe orthopedic injuries, compartment syndrome, hypotension, and organ damage (including acute kidney disease) are among the potential outcomes [8]. The systemic sign of a serious, traumatic muscular injury is the crushing syndrome. Continuous, persistent pressure on the limbs results in crush damage. The limb muscles are the main area of damage. Fasciotomy should be avoided, and conservative treatment methods should be used. To prevent infection of the wounded limb, radical debridement of the affected muscle should be conducted after fasciotomy, if performed. Infection is today's leading cause of morbidity and mortality, endangering the patient's life [9]. Compartment syndrome is one of the most dangerous side effects of a crush injury. It is crucial to prevent this situation or, at the very least, identify it early because it may result in irreparable harm and high morbidity. Treatment for a crush injury must include stabilizing fractures, mending soft tissue damage, and covering skin loss as soon as possible [10].

Depending on the injury, where it hits, and how severe it is, there are surgical and alternative treatments for managing midfoot injuries. Another aspect of surgery is if the harm caused is in the foot's weight-bearing area [11]. The essential role of the midfoot for weight-bearing function and its relation to the front and rear of the foot highlights the meaning of properly managing midfoot injuries. It is necessary to prove that the patient is capable of walking with a somewhat normal stride [12]. Physiotherapy (PT) in foot crush injury focuses on functions on the lower limb using interventions such as strengthening exercises, stretching, passive movements with several repetitions, and gradually increasing complexity. This is a case report of a 58-year-old male with a crush injury of the left medial aspect of his foot, and the goal of this paper is to prove the importance of PT in this condition, which is a common occurrence.

## Case presentation

Patient information

A 58-year-old male presented to Acharya Vinoba Bhave Rural Hospital, Wardha, Maharashtra, India, casualty with a lacerated wound on his right foot following a crush injury caused by the overturning of a bus tire on September 23, 2023. The patient experienced acute onset pain, tenderness, and discomfort exacerbated by movement. Examination revealed a 20-cm open wound on the medial aspect of the foot. The pain was aggravated with movement and was relieved at rest. Imaging confirmed fractures of all cuneiforms and cuboid bones, along with heel pad avulsion. Subsequently, the patient underwent wound debridement and a K-wire (Kirschner wire) operation for stabilization on October 7, 2023. Postoperatively, the patient complained of severe pain and numbness and was referred to musculoskeletal physiotherapy for further management.

Clinical findings

**Figure 1:**
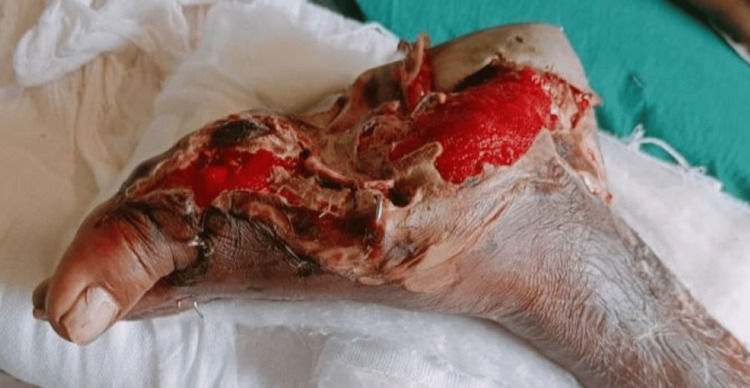
Image showing a lacerated wound on the medial aspect of the foot

The patient's written and verbal consent was obtained before conducting the physical examination. The patient was afebrile and hemodynamically stable. On examination, the patient was observed in a supine position. The patient had a 20-cm lacerated wound on the medial aspect of the foot, accompanied by swelling over the ankle and distal leg (Figure 1). Additionally, heel pad avulsion was observed. The patient rated his pain as 8/10 on a numerical pain rating scale. Palpation revealed localized warmth and further tenderness (grade 4) on the dorsum of the foot, while sensation was notably absent throughout the entire foot. 

Surgical intervention

The surgical intervention consisted of two distinct procedures. The initial operation involved the meticulous debridement of the wound, followed by the insertion of a K-wire for stabilization. Subsequently, a second procedure was performed, solely dedicated to the debridement of the wound associated with the compact grade 3 bone fracture. This comprehensive approach aimed to address both the wound care and the specific needs of the bone fracture, ensuring optimal patient recovery and healing.

Diagnostic assessment

**Figure 2:**
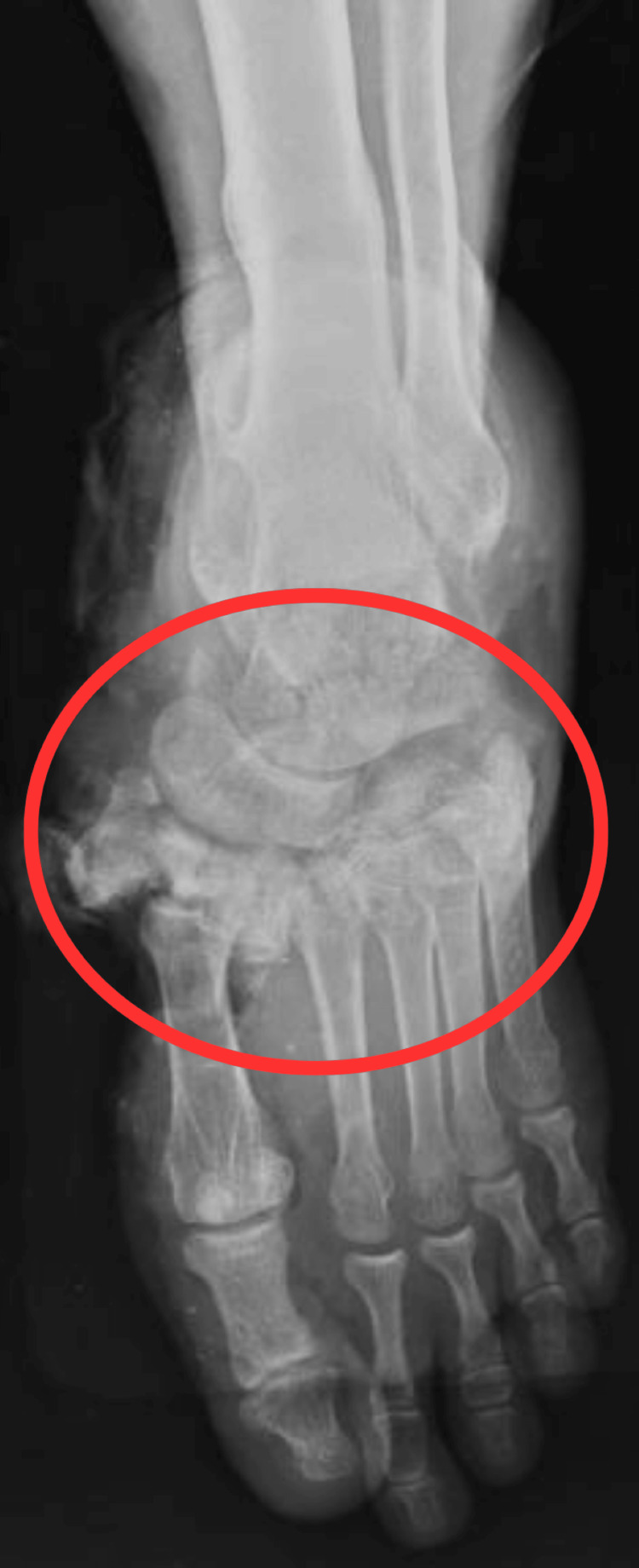
AP view of X-ray of the affected foot The circle shows fractures of the cuneiform and cuboid bone AP, anteroposterior

**Figure 3:**
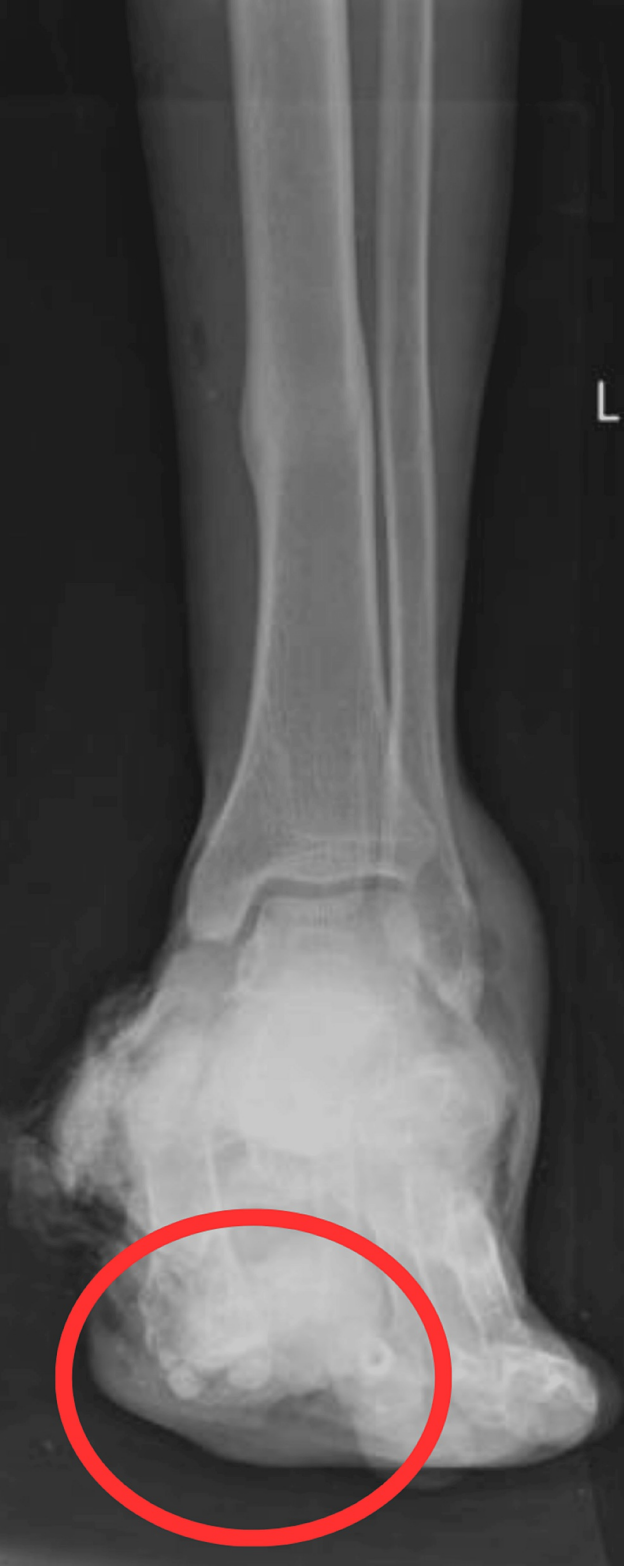
PA view of X-ray of the affected foot The circle shows the heel pad avulsion PA, posteroanterior

The patient underwent clinical and radiological examination, which revealed a Lisfranc fracture, cuneiform and cuboid fracture of the left foot, and heel pad avulsion of the left foot. Figure 2 shows an X-ray of the affected foot with cuneiform and cuboid fractures on the left side. Figure 3 shows an X-ray of the affected foot with heel pad avulsions on the left side.

Physiotherapy intervention

A four-week PT rehabilitation protocol was devised. Patient education aimed at informing the patient and his family about the patient's condition, including what steps to take to prevent complications and how crucial PT is to the patient's recovery. The patient was counselled regarding the value and impact of exercise, walking, and posture.

Patient Education

Patient education played a pivotal role in the rehabilitation program. The primary goal was to provide comprehensive information to the patient and his family regarding the patient's condition. This included guidance on preventive measures to avoid complications and emphasized the critical role that PT played in facilitating the patient's recovery. Counseling sessions were conducted to underscore the importance and impact of specific elements in the rehabilitation process. Emphasis was made on the significance of ambulation in rehabilitation, with guidance on progressively increasing walking duration. Education was provided on maintaining optimal posture during daily activities to promote musculoskeletal health and prevent further issues.

Week 1

During week 1 of the treatment, the PT rehabilitation protocol incorporated various exercises to address the patient's condition. Active range-of-motion (AROM) exercises targeted the unaffected limb, encompassing ankle-toe movements and knee flexion/extension. These exercises were performed in two sets of 10 repetitions for each joint. Isometric exercises focused on the quadriceps and hamstrings were integrated, involving three sets of 10-second holds with a gradual increase in intensity. Strengthening exercises for the upper limb comprised using a half-liter bottle, with three sets of 10 repetitions for each arm. The bridging exercise, involving three sets of 10 repetitions and a five-second hold in the bridge position, was implemented to enhance core stability.

Additionally, ambulation was introduced, emphasizing assisted non-weight-bearing walking with a walking aid. The ambulation sessions comprised short distances with a gradual progression, targeting five to 10 minutes, and recommended two to three times a day. Deep breathing exercises were taught to the patient to improve and maintain functional capacity. This multifaceted approach aimed to initiate the patient's recovery while considering his individual needs and progress.

Week 2

In the subsequent phase of the treatment, AROM exercises were introduced for the opposite limb, engaging in movements that targeted joint flexibility. Isometric exercises concentrated on the quadriceps and hamstrings, involving controlled muscle contractions. Strengthening exercises for the upper limb featured the use of a half-liter bottle, with a shift to three sets of 12 repetitions for each arm to further enhance upper body strength. The pelvic bridging exercise underwent refinement, with increased hold time and the incorporation of gentle passive movement. This exercise regimen comprised three sets of 12 repetitions, with participants maintaining the bridge position for eight seconds. To address mobility and coordination, continued non-weight-bearing assisted walking sessions were implemented. This ambulation practice involved gradual increments in duration, reaching 10-15 minutes, and was recommended for two to three sessions per day. This progressive approach aimed to optimize the rehabilitation process, adapting to the patient's evolving capabilities and ensuring a comprehensive recovery.

Week 3

In the subsequent phase of the treatment, the rehabilitation protocol evolved to encompass range-of-motion exercises tailored to the patient's progress. Active movement exercises were introduced for the opposite limb, with participants engaging in three sets of 10 repetitions each, performed three times a day to promote flexibility and joint mobility. Simultaneously, isometric exercises targeting the quadriceps and hamstrings were integrated, involving three sets of 10 repetitions each, conducted three times daily to enhance muscle strength and endurance. The rehabilitation program included dynamic quadriceps exercises, performed in three sets of 12 repetitions, contributing to functional movement patterns. Strengthening exercises for the upper limb featured the use of a half-liter bottle, with participants completing three sets of 12 repetitions for each arm, emphasizing upper body strength. The pelvic bridging exercise underwent modification, incorporating active assisted movement of the unaffected leg. This exercise regimen comprised three sets of 12 repetitions, integrating active assistance to enhance core stability and coordination. To address weight-bearing capabilities, ambulation was introduced, focusing on partial weight-bearing walking under close supervision. Sessions aimed for 15-20 minutes were recommended two to three times a day.

Week 4

In the final phase of the treatment, the rehabilitation protocol saw a continuation of AROM exercises tailored for the unaffected leg. The patient was engaged in exercises, focusing on joint flexibility and mobility. Strengthening exercises were intensified to encompass both upper and lower limbs. For the upper limb, individuals completed three sets of 12 repetitions for each arm, promoting comprehensive strength and endurance. Concurrently, the lower limb exercises progressed to weight-bearing activities, with the patient undertaking three sets of 12 repetitions to enhance lower extremity strength. To further advance the rehabilitation process, independent walking was introduced with close supervision during ambulation sessions. The patient was encouraged to walk independently for 15-20 minutes, two to three times a day, marking a significant milestone in restoring functional mobility. This comprehensive approach aimed to maximize the patient's recovery by addressing both upper and lower limb strength and promoting independent ambulation under supervision.

Follow-up and outcome measures

An organized physical therapy intervention procedure was started. There was a follow-up every week for four weeks. Table 1 shows the pre- and post-intervention range of motion, and Table 2 shows the outcome measures.

**Table 1:** Pre- and post-treatment range of motion NA, not applicable

Joint	Pre-rehabilitation	Post-rehabilitation
Hip flexion	0°-80°	0°-100°
Hip extension	0°-10°	0°-20°
Hip adduction	0°-15°	0°-30°
Hip abduction	0°-30°	0°-45°
Hip external rotation	0°-30°	0°-45°
Hip internal rotation	0°-30°	0°-45°
Knee flexion	0°-80°	0°-110°
Knee extension	NA	0°-10°
Ankle dorsiflexion	NA	0°-10°
Ankle plantarflexion	NA	0°-25°

**Table 2:** Outcome measures

Scales	Pre-rehabilitation	Post-rehabilitation
Numerical pain rating scale	8/10	3/10
Lower extremity functional scale	30/100	50/100

## Discussion

PT is essential for reducing pain, increasing range of motion, strengthening muscles, and improving hand function. It also lowers the chance of contractures, deformities, and stiffness following crush injuries. In this case, the patient visited the Department of Physiotherapy following a foot crush injury with complaints of pain, reduced range of motion, and challenges in doing activities of daily living. After evaluation, a four-week treatment plan was made, which included the use of the range-of-motion exercises. Brown et al. suggested that PT is effective in increasing the range of motion in the foot [13]. This study aimed to show how crush injury patients can benefit from physical therapy rehabilitation [14]. It outlines the injuries observed and how they were treated. Foot crush injuries are a relatively unusual emergency department presentation. The most frequent mechanism of injury involved a weight drop onto the foot, resulting in soft tissue damage in most patients. Even though crush injuries are rare, almost 25% of patients needed surgical intervention to treat their injuries, and one-third of patients needed hospitalization [15]. Nearly 25% of patients complained of persistent foot issues during the final review in the outpatient clinic, suggesting that even though these injuries are uncommon, they have the potential to increase morbidity and burden local orthopedic departments significantly. Compared to patients with soft tissue injuries alone, those with bone injuries after a crushing mechanism were much more likely to have persistent issues [16,17].

The tendons, ligaments, and muscles in the foot can all become more mobile by seeking treatment from a physical therapist [18]. The patient responded well to the treatment strategy; we were able to expand the patient's range of motion and enable him to carry out everyday tasks. It helped patients get back on track for a sustained recovery. The multidimensional approach of our PT intervention in this particular case has helped in reducing pain, restoring function, and preventing long-term complications, ultimately contributing to a better quality of life in the affected individuals [19]. PT expedites the healing process, but it can also enhance the overall standard of recovery. More specifically, injuries from crushed feet that are not treated by a physical therapist have a higher chance of healing improperly and taking longer to heal. The strength of this case is that the PT rehabilitation was initiated just after the postoperative period. Follow-up was not possible after the intervention, thus limiting the study [20].

## Conclusions

This study’s findings affirm the pivotal role of physical treatment in alleviating pain, enhancing range of motion, increasing muscular strength, and improving leg function while mitigating the likelihood of stiffness, contracture, and deformity in crush injury. It is advised that for such severe injury, tailored long-term follow-up and intensive physical therapy are crucial steps toward restoring functional ranges and strength. Subsequent investigations could focus on optimizing surgical techniques and exploring novel rehabilitation approaches to improve outcomes in foot crush injuries.
